# Exploring Changes in the Host Gut Microbiota During a Controlled Human Infection Model for *Campylobacter jejuni*

**DOI:** 10.3389/fcimb.2021.702047

**Published:** 2021-08-31

**Authors:** Blake W. Stamps, Janelle Kuroiwa, Sandra D. Isidean, Megan A. Schilling, Clayton Harro, Kawsar R. Talaat, David A. Sack, David R. Tribble, Alexander C. Maue, Joanna E. Rimmer, Renee M. Laird, Chad K. Porter, Michael S. Goodson, Frédéric Poly

**Affiliations:** ^1^711th Human Performance Wing, Air Force Research Laboratory, Wright-Patterson Air Force Base, Dayton, OH, United States; ^2^Integrative Health and Performance Sciences Division, UES, Inc., Dayton, OH, United States; ^3^Enteric Diseases Department, Naval Medical Research Center, Silver Spring, MD, United States; ^4^Henry M. Jackson Foundation for the Advancement of Military Medicine, Bethesda, MD, United States; ^5^Center for Immunization Research, Johns Hopkins Bloomberg School of Public Health, Baltimore, MD, United States; ^6^Infectious Disease Clinical Research Program, Preventive Medicine and Biostatistics Department, Uniformed Services University of the Health Sciences, Bethesda, MD, United States; ^7^Academic Department of Military Medicine, Royal Centre for Defence Medicine, Medical Directorate, Joint Medical Command, Information and Communications Technology Centre, Birmingham, United Kingdom

**Keywords:** *Campylobacter jejuni*, microbiota, diarrhea, dysbiosys, human infection model

## Abstract

*Campylobacter jejuni* infection is a leading cause of foodborne disease, common to children, adult travelers, and military populations in low- to middle-income countries. In the absence of a licensed vaccine, efforts to evaluate prophylactic agents are underway. The prophylactic efficacy of a twice-daily, 550 mg dose of the antibiotic rifaximin demonstrated no efficacy against campylobacteriosis in a controlled human infection model (CHIM); however, samples from the CHIM study were utilized to assess how the human gut microbiome responds to *C. jejuni* infection, and if a ‘protective’ microbiota exists in study participants not developing campylobacteriosis. Statistically significant, but minor, differences in study participant beta diversity were identified during the challenge period (p = 0.002, R^2^ = 0.042), but no significant differences were otherwise observed. Pre-challenge alpha diversity was elevated in study participants who did not develop campylobacteriosis compared to those who did (p < 0.001), but alpha diversity declined in all study participants from the pre-challenge period to post-discharge. Our work provides insight into gut microbiome shifts observed during a *C. jejuni* CHIM and following antibiotic treatment. This study utilized a high dose of 1.7 x 10^5^ colony-forming units of *C. jejuni*; future work could include CHIM studies performed with inocula more closely mimicking natural exposure as well as field studies involving naturally-occurring enteric infections.

## Introduction

*Campylobacter jejuni* is a common, potentially commensal member of the avian gut microbiome (Hermans et al., 2012; Humphrey et al., 2014) as well as all warm-blooded animals and the food derived from their tissue; yet, when introduced into the human gut *via* the ingestion of contaminated food, water, or direct contact with animals, it can cause acute illness as it colonizes the human gut (Coker et al., 2002; Gölz et al., 2014; Kaakoush et al., 2015). *C. jejuni*-attributed illness, campylobacteriosis, is typified by mild to severe diarrhea with and without blood and/or mucous and fever. *C. jejuni* is a leading cause of foodborne disease, associated with 7.5 million disability-adjusted life years globally (Murray et al., 2012, 1990–2010). *C. jejuni* infection is also associated with the development of Guillain-Barré syndrome, reactive arthritis, and other autoimmune disorders (Mishu and Blaser, 1993; Garg et al., 2008; Malik et al., 2014). Significant mortality and morbidity in pediatric populations in low- to middle-income countries (LMICs), and are also caused by adult travelers to those same regions, is also caused by *C. jejuni*-attributed illness (Elaine  Scallan et al., 2011; Platts-Mills et al., 2015; Olson et al., 2019). One group of travelers that is often at risk for campylobacteriosis, as well as other enteric diseases, is deployed military (Riddle et al., 2006). In fact, acute diarrhea is the leading infectious disease threat to deployed U.S. forces (Riddle et al., 2017). A recent study of deploying Air Force personnel noted that over 50 percent of study participants who deployed to the Middle East had at least one diarrheal incident while on deployment, consistent with multiple systematic reviews highlighting a high incidence of diarrhea in deploying populations (Riddle et al., 2006; Porter et al., 2017b; Olson et al., 2019; Stamps et al., 2020). One of the leading etiologies of diarrhea in military populations is *C. jejuni*, accounting for approximately 10% of all acute diarrheal illness (Connor et al., 2012).

Reducing the risk of *C. jejuni* in military populations, and indeed in all at-risk populations, is desirable. In the absence of a licensed vaccine, efforts to develop safe and effective prophylactic agents targeting *C. jejuni* are underway. One method that enables an early assessment of prophylactics is the controlled human infection model (CHIM), in which a known dose of a well-characterized organism is administered to susceptible study participants in a controlled environment (Darton et al., 2015; Porter et al., 2017a). The *C. jejuni* CHIM is well-established (Poly and Guerry, 2008; Tribble et al., 2009; Kirkpatrick et al., 2013), and was used to previously assess the prophylactic efficacy of a twice-daily, 550 mg dose of rifaximin (Rimmer et al., 2018). Although rifaximin did not prevent campylobacteriosis, samples collected during the study afforded the opportunity for a novel evaluation of the host microbiome in response to chemoprophylaxis, *C. jejuni* challenge, and subsequent antibiotic treatment.

The human gut microbiome is known to meaningfully influence health and well-being, and our knowledge of its microbial communities and their functional capacity is expanding rapidly (Guinane and Cotter, 2013). Adaptive host metabolism, physiology, nutrition, and immune function have all been linked to these complicated communities (Guinane and Cotter, 2013). Conversely, long-term disruption of the gut microbiota (or dysbiosis) has been linked to a number of pathological and systemic diseases (Ley et al., 2006; Frank et al., 2007; Zhang et al., 2009; Kau et al., 2011; Qin et al., 2012; Guinane and Cotter, 2013; Candela et al., 2014), although the exact definition of what constitutes a dysbiotic state is controversial (Brüssow, 2020). Independent of the exact link between disease and dysbiosis, the gut microbiota can also confer protection (or colonization resistance) against enteric pathogens (Buffie and Pamer, 2013); nevertheless, this resistance can be impaired by even a short course of antibiotic treatment through a loss of beneficial species and/or an increase in antimicrobial resistance among strains (Buffie and Pamer, 2013; Rashid et al., 2015; Becattini et al., 2016; Fodor et al., 2018; Jørgensen et al., 2019). While the microbial community is capable of recovering (*i.e.*, eubiosis) to its normal state following such dysbiosis, the degree and/or timing of community composition recovery is variable (Modi et al., 2014). Disruptions to the gut microbiota can be induced by dietary shifts, consumption of antibiotics, as well as invasion by pathogens, acting either indirectly (*e.g.*, travel) or directly [*e.g.*, antibiotics, pathogen colonization, (David et al., 2014; Francino, 2016)]. While identifying a global definition of dysbiosis is beyond the scope of this work, herein, we define a dysbiosis as a significant shift in microbial community composition following the controlled introduction of either antibiotics or a pathogen. In the case of the pathogen, this is suggestive of both the successful colonization of *C. jejuni* and the induction of campylobacteriosis (*i.e*, infection) in the host. Further, dysbiosis may result from the antibiotics used to treat the *C. jejuni* infection at the end of the challenge period. In this way, our CHIM offers the ability to study three potential dysbiosis-inciting events: antibiotic prophylaxis with rifaximin, challenge with *C. jejuni*, and administration of dual antibiotic treatment with ciprofloxacin and azithromycin. Previous CHIM studies focusing on enterotoxigenic *Escherichia coli* (ETEC) and norovirus and the human gut using either 16S rRNA gene sequencing or metagenomics exist; however, none have specifically targeted the changes in the human gut microbiome during *C. jejuni* infection (Pop et al., 2016; Patin et al., 2020).

In this study, we explored the gut microbiota of 28 volunteers prior to and during rifaximin chemoprophylaxis, during *C. jejuni* strain CG8421 challenge, and following antibiotic treatment and elimination of the challenge organism. We sought to assess whether any of these insults to the microbiome were associated with microbial dysbiosis, and, if so, whether the gut microbiome returned to its pre-challenge state. We also examined whether any significant differences in the microbial diversity (*i.e.*, alpha diversity) of study participants previously enrolled in non-*Campylobacter* CHIM studies could be observed. Finally, we sought to identify a specific pre-challenge microbiome profile that modulated host susceptibility (*i.e.*, a microbiome that was predictive of infection) to campylobacteriosis.

## Methods

Metadata and stool samples from our previously published *C. jejuni* CHIM were utilized (Rimmer et al., 2018). A summary of the major outcomes of the Rimmer et al. study is provided in **Table 1**, and a brief description of the study design is outlined below. The clinical trial from which data and samples were obtained was registered with ClinicalTrials.gov on 29 October 2014 (NCT02280044), and was reviewed and approved by the Naval Medical Research Center and Western Institutional Review Boards in compliance with all applicable local, federal, and Department of Defense regulations governing the protection of human study participants. The protocol under which this work was performed (IDCRP-079) was reviewed and approved by the Institutional Review Board at the Infectious Disease Clinical Research Program (IDCRP). All study participants consented to allow for their feces to be sequenced as outlined below.

**Table 1:** Summary of major findings, adapted from (Rimmer et al., 2018).

Endpoint	Placebo (n = 13)	Rifaximin (n = 15)
Campylobacteriosis	11 (84.6)	13 (86.7)
Dysentery	4 (30.8)	4 (26.7)
Abdominal pain or cramps	9 (69.2)	11 (73.3)
Nausea	5 (38.5)	6 (40.0)
Vomiting	0 (0.0)	3 (20.0)
Fever	6 (46.2)	7 (46.7)
Total No. of loose stools, median (Q1, Q3)	18 (11, 23)	12 (7, 18)
Maximum No. of loose stools in 24 hours, median (Q1, Q3)	9 (8, 11)	7 (3, 12)
Duration of diarrhea, hours, median (Q1, Q3)	100.4 (83.3, 115.2)	75.2 (71.2, 113.6)
Campylobacteriosis score, median (IQR)	8 (6, 10)	7 (6, 10)
Study participant recrudescence	3	2
Total 16S samples	131	157

### Study Description

**Figure 1:**
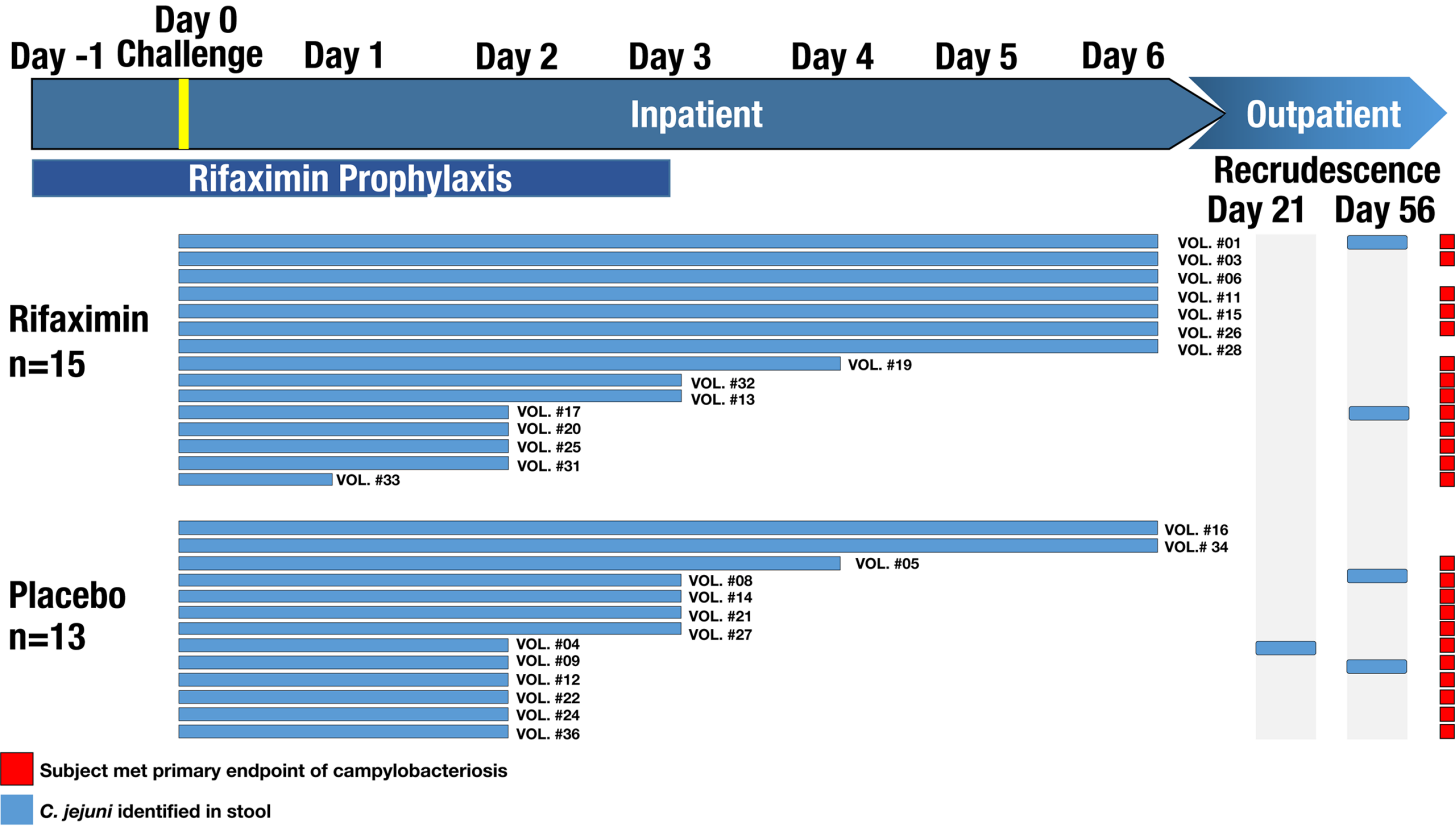
Study design conceptual overview. The length of each study participant (SID) bar shows when *C. jejuni* was identified in their stool. Red squares denote which study participants met the endpoint of the study (*i.e.*, campylobacteriosis).

A randomized, double-blind, placebo-controlled study was conducted to assess the efficacy of rifaximin chemoprophylaxis against campylobacteriosis in a CHIM. Healthy adults from the mid-Atlantic area with no history of *Campylobacter* infection, personal or family history of Guillain-Barré syndrome, inflammatory arthritis, or who were positive for HLA-B27 were included in the study. Prior *Campylobacter* exposure was determined using serum immunoglobulin A (IgA) titer to *C. jejuni *CG8421 glycine extract (GE) >1:4,000 as described previously (Rimmer et al., 2018). Study participants were admitted to an inpatient facility and randomized 1:1 to either a 550 mg twice-daily dose of rifaximin or placebo (**Supplemental Table S1**). Study participants began prophylaxis the day prior to challenge (study day -1, **Figure 1**) and continued for 4 days unless they met the primary endpoint of campylobacteriosis. Additional information on study participants, including age, sex, and symptoms during the CHIM can be found in **Supplemental Table S1**. Additionally, whether or not a study participant was previously enrolled in other CHIMs can be found in **Supplemental Table S1**.

On the day of challenge (study day 0), study participants received 1.7 x 10^5^ colony-forming units of the *C. jejuni* strain CG8421 following a light breakfast and 90-minute fast. The primary clinical endpoint was campylobacteriosis within 144 hours of challenge, defined as: moderate to severe diarrhea (≥4 loose stools or ≥401 g of loose stools in 24 hours) or fever (oral temperature ≥38.0°C), with an associated symptom of nausea, vomiting, abdominal cramps, tenesmus, or gross blood in at least 2 stools. Fluid status was closely monitored and rehydration therapy was administered orally or intravenously, as clinically appropriate. All study participants were treated with ciprofloxacin (500 mg twice daily) concurrently with azithromycin (500 mg once daily) for 5 days starting approximately 144 hours following challenge or upon meeting early treatment criteria. Study participants were discharged when they were no longer shedding the challenge strain and their symptoms had resolved or were resolving. Post-discharge clinic visits with stool culture occurred approximately 14, 21, 28, 35, 56, and 84 days following challenge. Stool samples were collected in 50 mL Falcon tubes and placed on dry ice until being stored at -80°C. Notably, 2 study participants from the rifaximin group (on day 56) and 3 study participants from the placebo group (1 on day 28, and 2 on day 56) presented with the challenge strain in their stool post-discharge. All study participants were asymptomatic, and were re-treated with dual antibiotic therapy (ciprofloxacin and azithromycin for longer courses). One study participant experienced two recrudescent events, first on day 21 and then on day 56, and was started on amoxicillin-clavulanate for 7 days with a probiotic on day 56.

### DNA Isolation and 16S rRNA Gene Library Preparation

A total of 46 pre-challenge stools, 197 stools collected during the inpatient study phase, and 52 stools collected during the post-discharge study phase were selected for DNA extraction. Metadata used for analysis of the dataset are included as **Supplemental Table S1**. Frozen stool samples (100 mg to 200 mg) were subjected to whole DNA extraction using the PowerSoil DNA Isolation Kit (QIAGEN N.V., Hilden, Germany) following the manufacturer’s protocol. DNA concentration was estimated using a NanoDrop spectrophotometer (Thermo Fisher Scientific, Waltham, MA) for all samples. Extracted, quantified nucleic acids were stored at -20°C until further use.

Amplification and preparation of 16S rRNA gene sequencing libraries were performed following the Illumina 16S rRNA gene protocol (Illumina Part Number 15044223 Rev. B) according to the manufacturer’s instructions. Briefly, the 16S rRNA gene was amplified using the 16S-specific primers described previously (Klindworth et al., 2013): 519F (5’TCGTCGGCAGCGTCAGATGTGTATAAGAGACAG **CCTACGGGNGGCWGCAG**) and 785R (5’GTCTCGTGGGCTCGGAGATGTGTATAAGAGACAG **GACTACHVGGGTATCTAATCC**), where the 16S rRNA gene-specific region is bolded, and the Illumina-specific overhangs are underlined. These primers amplify a product of ≈ 460 base pairs (bp) that contains the V3 and V4 regions of the 16S rRNA gene. The library was then generated using Illumina sequencing adapters and dual‐index barcodes. Sequencing of the 16S library was performed on an Illumina MiSeq sequencer (Illumina Inc., San Diego, CA) using V3 PE300 chemistry at the University of Wisconsin Biotechnology Center.

### Sequence Analysis

De-multiplexed (*i.e.*, per-sample), paired FASTQ files were returned from the sequencing facility. Quality control, trimming, and amplicon sequence variant (ASV) clustering were carried out using DADA2 (Callahan et al., 2016, 2) within the R version 4.0.2 programming environment (R Core Team, 2013). After visualizing a subset of sequence reads using DADA2 to establish quality thresholds, forward and reverse reads were truncated to 280 bp and 230 bp, respectively. A 30 bp region from the 5’ of each read was also truncated to ensure the complete removal of the PCR primer. Reads were also screened to remove PhiX and a maximum expected entropy (maxee) of 2 was allowed. Chimeric sequences were removed using the consensus method within DADA2, after which ASV taxonomy was assigned using the DADA2 classifier against the SILVA r132 database (Quast et al., 2013). An alignment of ASVs was produced using the IPS package within R, and a tree was produced using FastTree2 (Price et al., 2010). Data were then imported into the Phyloseq and Ampvis2 packages (McMurdie and Holmes, 2013; McMurdie and Holmes, 2014; Andersen et al., 2018, 2) for statistical analysis and visualization. Prior to analysis, any sequence reads classified as ‘Eukaryota’, ‘Chloroplast’, or ‘Mitochondria’ were removed. For all statistical analyses, a *Campylobacter*-dominated sample from study participant 8 (who had multiple asymptomatic recrudescence events and a sample in which the genus *Campylobacter* represented over 90 percent relative abundance of all detected taxa) was removed as an extreme outlier. All other samples from study participant 8 were retained for analyses.

The microbiota from this cohort was compared to that described from two separate studies among a comparable population to ensure our study population was representative of the broader population. To this end, rRNA gene sequence data from 1,077 samples downloaded from the Broad Institute-OpenBiome Microbiome Library (BIO-ML) 16S rRNA gene sequence library (Poyet et al., 2019) were clustered into ASVs alongside the samples from our study. Briefly, clustering was performed as above for each dataset separately, with DADA2 trimLeft parameters set as (195,76) and (21,43) for our dataset and the BIO-ML dataset, respectively. The trim options were set to ensure an overlapping sequence space, as the primer sets varied slightly for each dataset (position 341 to 785, and 515 to 806, respectively). After clustering within DADA2, sequence tables were merged and, to reduce the bias from the use of differing primer sets, ASVs were clustered using the ‘tip_glom’ function within Phyloseq and a cophenetic distance of 0.1. Differences between BIO-ML fecal samples and those taken within the CHIM were then visualized by principal component ordination using a weighted UniFrac distance matrix within Ampvis2 (Lozupone and Knight, 2005).

Alpha diversity was assessed using the R packages DivNet and Breakaway (Willis and Bunge, 2015; Willis et al., 2017; Willis and Martin, 2020). Briefly, Shannon diversity was calculated for each sample within DivNet, and then tested for significance using the betta function within Breakaway. Beta diversity was assessed by a permutational analysis of variance within R using the adonis function (Anderson, 2001; Dixon, 2003). For both alpha and beta diversity tests, a p-value greater than 0.05 was reported as ‘p > 0.05’ and any value less than 0.0001 was reported as ‘p < 0.0001’. A Permutational Multivariate Analysis of Variance Using Distance Matrices (commonly referred to as an ‘adonis’ test, so named after the R function) was run within the R package Vegan to assess significant differences in beta diversity between test groups. For adonis test results, any significant test also had its R^2^ value reported. A corresponding homogeneity of dispersion test (betadisper) was also run for each tested factor. Microbial taxa that were significantly, differentially abundant were identified using corncob (Martin et al., 2020). As a secondary confirmatory method, we used random forests to identify microbial taxa potentially predictive of reducing campylobacteriosis risk (Breiman, 2001; Liaw and Wiener, 2002). The 50 ASVs responsible for the greatest mean Gini decrease are reported.

## Results

**Figure 2:**
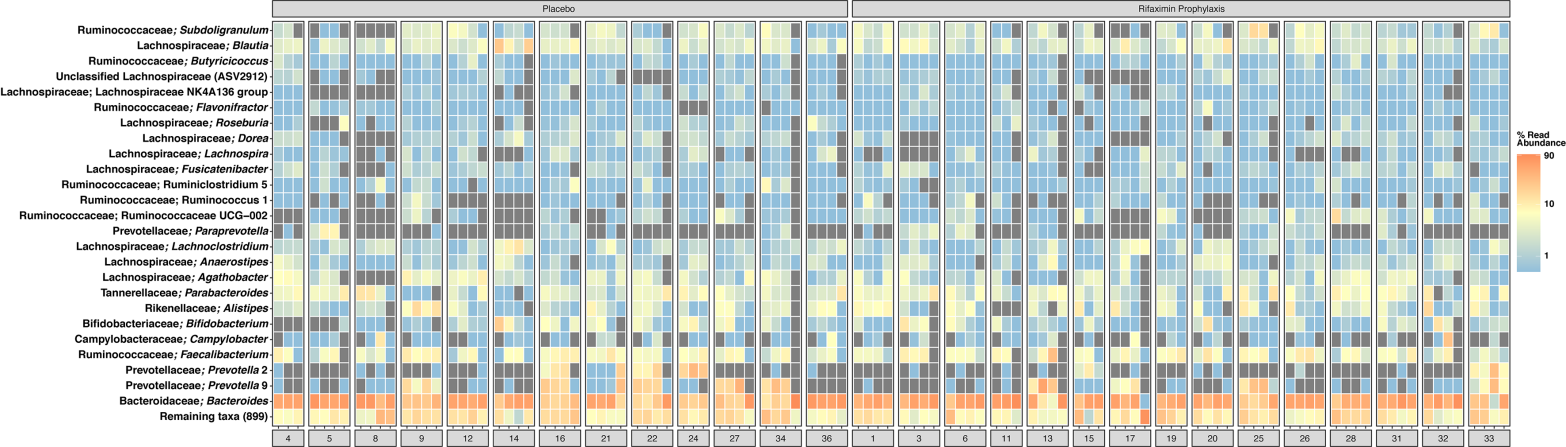
Heatmap of the top 50 most abundant amplicon sequence variants (ASVs), clustered at the level of species if possible. Taxonomy shown is the identified genus, and the most closely related species, if known. Samples are averaged into primary phases of the CHIM for each study participant, including pre-challenge, inpatient, post-ciprofloxacin/azithromycin administration, and post-discharge. Samples are shown faceted by treatment group; among those study participants either receiving placebo or rifaximin prophylaxis. Gray squares indicate an ASV was not detected. For clarification on sample phase definitions (pre-challenge, inpatient, post-challenge, etc.), please see **Figure 1**. Heatmap scale is log_10_.

A total of 11,708,067 sequence reads were clustered into 4,059 ASVs from 198 samples, with a mean library size of 61,117 sequence reads. Further descriptive statistics of sequence data are available in **Supplemental Table S2A**, and a rarefaction curve of all samples is shown in **Supplemental Figure S1**. The beta diversity (*i.e.*, the microbial community composition) of baseline samples was similar to publicly available American/western gut microbiomes within the BIO-ML database when visualized by principal coordinate ordination (**Supplemental Figure S2**). Prior to challenge, study participants had stool samples composed predominantly of ASVs most closely related to the genera *Bacteroides, Faecalibacterium, Prevotella*, and other common human gut commensal bacteria (**Figure 2**). More specifically, *Bacteroides vulgatus* (11.2% average relative abundance), *Bacteroides uniformis* (5.0% average relative abundance)*, Bacteroides stercoris* (3.2% average relative abundance), *Faecalibacterium prausnitzii* (3.1% average relative abundance), *Blautia* (1.5% average relative abundance), *Parabacteroides merdae* (3.2% average relative abundance), and, in a smaller subset of study participants, *Prevotella* (4.0% average relative abundance) were common during the pre-challenge, inpatient, and post-antibiotic treatment administration periods (**Supplemental Figure S3**). *Campylobacter* was identified in low, but detectable relative abundances across all samples during the inpatient period (*i.e.*, post-challenge and prior to antibiotic treatment) (**Figure 2** and **Supplemental Figure S3**). When compared to pre-challenge samples, ASVs most closely related to *Faecalibacterium prausnitzii* were only intermittently abundant during the post-discharge period (**Supplemental Figures S3**, **S4**). A complete list of detected taxa is available in **Supplemental Table S2B**.

### Differences in Study Participant Microbiome Richness and Diversity

**Figure 3:**
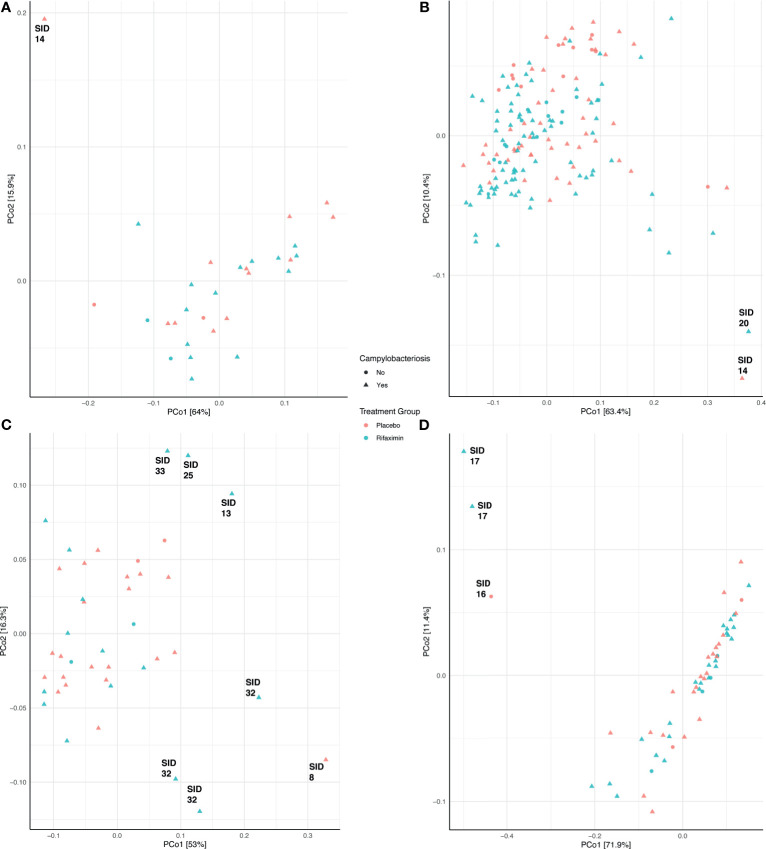
Principal coordinate analysis (PCoA) of samples during the pre-challenge **(A)**, inpatient **(B)**, post-antibiotic administration **(C)**, and post-discharge **(D)** periods. Each PCoA was generated from a weighted UniFrac distance matrix. Samples are colored by treatment group (red, placebo; blue, rifaximin), and sample shape denotes samples from study participants who either developed campylobacteriosis (triangles) or did not (circles).

Community composition was not significantly different (adonis p > 0.05) between treatment groups (placebo- or rifaximin-prophylaxed) during any period (**Figures 3A, C, D**), with the exception of the inpatient period (**Figure 3B**, adonis p = 0.001, R^2^ = 0.042). Pre-challenge samples among study participants who developed campylobacteriosis had significantly different (albeit not visually obvious) community composition (**Figure 3A**, adonis p = 0.044, R^2^ = 0.120), but not significantly different composition (adonis p > 0.05) during all other sampling periods (**Figures 3B–D**). When visualized by principal coordinate ordination, samples from study participants who did not develop campylobacteriosis were nested within samples from study participants who developed campylobacteriosis (*i.e.,* their community composition was a subset of that from those who did develop campylobacteriosis, **Figure 3B**). Microbial community composition was not significantly different between study participants with and without microbial recrudescence at any point in the study (adonis p > 0.05). While there was a significant difference in community composition between samples from the pre-challenge to the post-discharge periods (p = 0.038, R^2^ = 0.043), when visualized by principal coordinate ordination, differences appeared to be driven by a small number of outlying observations from the post-discharge period (**Supplemental Figure S5**). Outliers in PCoA ordination appeared driven by several taxa. Prior to the inpatient period, the one notable outlier (study participant 14) had a greater relative abundance of *Blautia massiliensis* and *Bifidobacterium longum* than other study participants (**Supplemental Figure S3**). During the inpatient period and after the administration of *Campylobacter* to all study participants, study participants 20 and 14 were notable outliers, with no clear shared taxa between the two study participants, although both had numerous *Bacteroides* ASVs in abundance (**Supplemental Figure S3**). In post-antibiotic administration samples, study participants 8 and 32 both had elevated relative abundances of *Campylobacter* remaining in their stool samples, whereas study participants 13, 25, and 33 had lower relative abundances of ASVs most closely related to *Campylobacter* and *Bacteroides uniformis*, and a corresponding increase in the abundances of ASVs most closely related to *Faecalibacterium prausnitzii* and *Subdoligranulum* (**Supplemental Figure S3**). Finally, in the post-discharge period, study participants 16 and 17 were notably lacking the majority of ASVs in abundance in all other study participants (**Supplemental Figure S3**).

**Figure 4:**
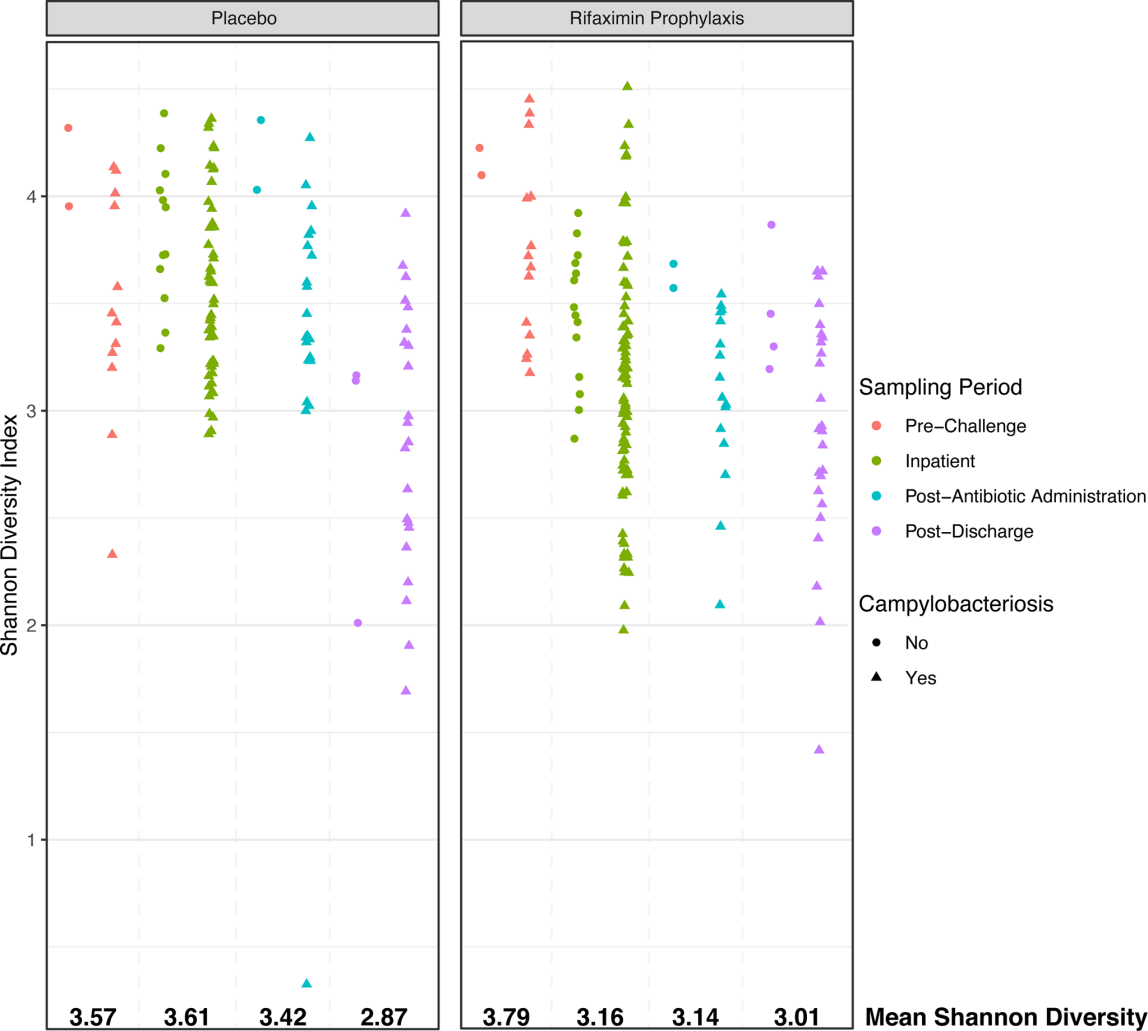
Shannon alpha diversity plot comparing placebo- and rifaximin-treated study participants during each phase of the CHIM. Sample shape denotes samples from study participants who either developed campylobacteriosis (triangles) or did not (circles).

While beta diversity was not significantly different between study participants other than during the inpatient period (albeit with a very low R^2^), there were significant differences in microbial diversity as assessed by the Shannon diversity index. In placebo and rifaximin recipients, Shannon diversity declined from pre-challenge to post-discharge periods (**Figure 4**, overall Shannon diversity mean: 3.80 pre-challenge, 3.08 post-discharge). While pre-challenge Shannon diversity was marginally different between the study groups (**Figure 4**, p = 0.049, mean: 3.57 placebo, 3.79 rifaximin), diversity was significantly higher (p < 0.001) in study participants who did not develop campylobacteriosis as compared to those who did, although sample numbers for study participants not developing campylobacteriosis (n = 4) were much lower than for those who did (n = 24). There were no significant differences in baseline samples (p > 0.05) in Shannon diversity among study participants who had participated in previous enteric CHIMs.

During the inpatient period, Shannon diversity was highly significantly different (p < 0.001) between those who did or did not develop campylobacteriosis, as well as between study participants who had or had not previously participated in enteric CHIMs. Specifically, Shannon diversity was lower in rifaximin recipients (Shannon diversity mean: 3.16) than placebo recipients (Shannon diversity mean: 3.62). Placebo recipients and those not developing campylobacteriosis had significantly higher Shannon diversity (**Figure 4**). Mean diversity in both treatment groups declined after antibiotic treatment (overall mean Shannon diversity = 3.30), and continued to decline in post-discharge samples (**Figure 3**, overall mean Shannon diversity = 3.08). Post-discharge, there were no significant differences in Shannon diversity between treatment groups (p > 0.05), but diversity was lower in those study participants who had developed campylobacteriosis (p = 0.028, **Figure 3**, mean Shannon diversity = 2.91) compared to study participants who had not (mean Shannon diversity = 3.16). In contrast to the significant differences in Shannon diversity observed during the inpatient period, there were no significant differences (p > 0.05) between study participants who had previously been enrolled in an enteric CHIM (Shannon diversity = 3.73) and those who had not prior to challenge (Shannon diversity = 3.62). Post-discharge, study participants who had previously been enrolled in an enteric CHIM (**Supplemental Table S1**) had significantly higher Shannon diversity (p = 0.004, mean Shannon diversity = 3.05) when compared to first-time CHIM participants (mean Shannon diversity = 2.74).

### Identification of Microbial Taxa Associated With Dysbiosis

**Figure 5:**
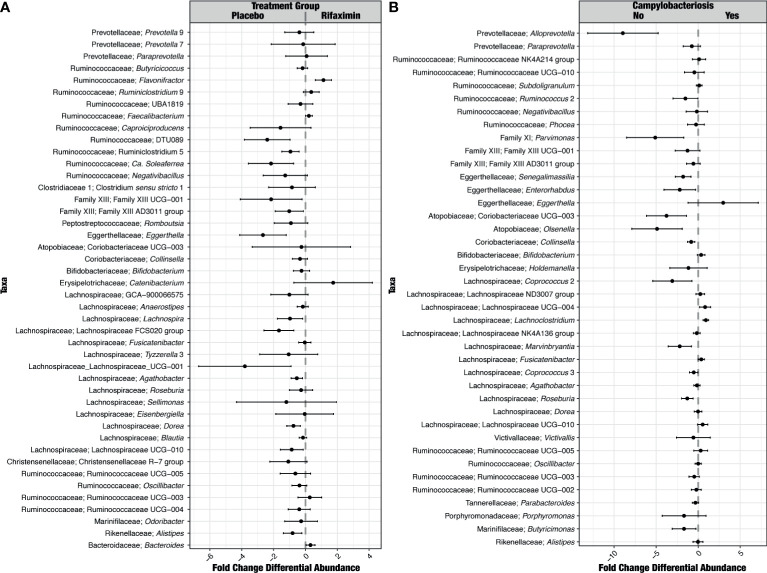
Identification of ASVs that were differentially abundant between treatment groups **(A)** and study participants who did or did not develop campylobacteriosis **(B)** during the inpatient period. For differences between treatment groups **(A)**, taxa to the right of the plot were significantly greater in relative abundance in study participants in the rifaximin arm of the study. For differences between study participants who did or did not develop campylobacteriosis **(B)**, taxa to the right of the plot were significantly more abundant in those study participants who did develop campylobacteriosis.

Very few taxa were differentially abundant between study groups pre-challenge. Differentially abundant taxa included three unclassified Lachnospiraceae genera (ND3007, NK4A136, and UCG-004) and *Parabacteroides* (**Supplemental Figure S6A**). Following *C. jejuni* challenge, there were far more differences by study group. *Mitsuokella* and Erysiplotrichaceae UCG-003 were greater than 2-fold differentially abundant in study participants receiving rifaximin; *Prevotella*, *Actinomyces*, *Rothia*, *Erysipelatoclostrium*, Lachnospiraceae UCG-001, and *Fusobacterium* were greater than 2-fold differentially abundant in placebo recipients (**Figure 5A**). While *Campylobacter* differential abundance was not significantly different between study groups during the inpatient period, it was significantly different and in greater relative abundance in study participants who experienced recrudescent *Campylobacter* infection (**Figure 5A**).

Very few taxa were differentially abundant in pre-challenge samples between study participants developing and not developing campylobacteriosis, although the high relative abundance genus *Bacteroides* was significantly higher (FDR p < 0.05) in study participants who developed campylobacteriosis, as was *Ruminiclostridium* group 6 (**Supplemental Figure S6B**). During the inpatient period, far more taxa were more significantly differentially abundant (FDR p < 0.05) among study participants without campylobacteriosis (**Figure 5B**), with only *Eggerthella* more than 2-fold more abundant in study participants with campylobacteriosis. The most differentially abundant taxa (> 2-fold) in study participants without campylobacteriosis post-challenge but prior to antibiotic treatment included *Alloprevotella*, Coriobacteriaceae UCG-003, *Olsenella*, *Parvimonas*, and *Coprococcus* (**Figure 5B**). Three taxa were significantly differentially abundant in pre-challenge samples for study participants who would go on to recrudesce after challenge - *Butyricioccus, Parasutterella*, and *Agathobacter*.

After antibiotic treatment, Lachnospiraceae UCG-010, UCG-004, and *Subdoligranulum* were significantly differentially abundant in study participants who had experienced campylobacteriosis. In study participants without campylobacteriosis, Ruminococcaceae NK4A214 and NK4A136, Clostridia Family XIII AD3011 group, and unclassified Ruminococcaceae UCG-005, UCG-002, and UCG-003 were significantly differentially abundant (**Supplemental Figure S6C**). During the post-discharge period, no taxa were significantly differentially abundant between those who did or did not have campylobacteriosis.

A random forest model produced an out-of-bag error rate of 1.74 percent, and zero error in classifying samples as coming from study participants with campylobacteriosis. Samples from study participants without campylobacteriosis had a class error of 12.2 percent, with 5 of 41 total samples classified incorrectly. While *Campylobacter* itself was not important for classifying samples as coming from study participants with or without campylobacteriosis, one ASV, most closely related to the unclassified Ruminococcaceae UCG-002, decreased model accuracy by 5.3 percent. Other potentially important predictors included unclassified *Bacteroides* and *Paraprevotella* (**Table 2**). A complete table of predictor importance is available in **Supplemental Table S3**.

**Table 2:** ASVs identified with greater than 1% effect in mean model accuracy decrease.

Predictor	Mean Decrease Gini (Accuracy)	Nearest Relative
ASV1	3.99	*Bacteroides*
ASV4256	3.51	*Bacteroides vulgatus*
ASV3793	2.98	Ruminococcaceae UCG-002
ASV243	2.66	*Paraprevotella*
ASV261	2.08	*Bacteroides massiliensis*
ASV4080	1.58	*Butyricimonas*
ASV104	1.51	*Bacteroides*
ASV4194	1.19	*Bacteroides vulgatus*
ASV4180	1.17	*Bacteroides*
ASV116	1.13	*Alloprevotella*
ASV922	1.04	Unclassified Ruminococcaceae
ASV115	1.03	*Alloprevotella*

ASV phylogeny is shown as the closest identified genus and, if identified, species.

## Discussion

Campylobacteriosis is a global public health problem to both adults and children even when not causing acute illness (Scallan et al., 2011; Murray et al., 2012; Platts-Mills et al., 2015), as well as a significant concern for deploying military populations (Connor et al., 2012; Olson et al., 2019). While antibiotic treatments exist, efforts to develop safe and effective prophylactics to prevent disease are currently underway; however, none are licensed for this purpose. A *C. jejuni* CHIM demonstrated that rifaximin chemoprophylaxis did not protect against campylobacteriosis (Rimmer et al., 2018). Nevertheless, that study provided a unique opportunity to explore how prophylactic antibiotics and subsequent challenge with *C. jejuni* induce dysbiosis in the human gut. By carefully tracking how the human gut microbiome shifts prior to challenge, during challenge, and after treatment, multiple microbial taxa were identified that may act to protect against enteric illness.

At baseline, the microbiome of our study participants was similar to other individuals consuming a westernized diet in the broader U.S. population (**Supplemental Figure S2**) (Poyet et al., 2019). More specifically, this baseline microbiome was defined by increased alpha diversity relative to post-discharge samples (**Figure 4**), high relative abundances of common gut commensal genera, such as *Bacteroides*, *Faecalibacterium*, and, within a smaller number of study participants, *Prevotella* (**Figure 2**). Very few ASVs were significantly differentially abundant between rifaximin and placebo recipients. Significantly differentially abundant taxa identified included uncultivated members of the *Lachnospiraceae*: Lachnospiraceae ND3007, NK4A136, and UCG-004. None were in high relative abundances in any sample (**Figure 2**), and it is likely they played little to no role in the observed differences in study participants upon challenge. While these differences between groups were apparently minor, future studies may consider pre-sequencing study participants’ fecal samples utilizing real-time sequencing technologies prior to group assignment to fully randomize study participants even at the level of taxonomic distributions in their microbiome. Upon *C. jejuni* challenge, larger differences between study participants emerged which most likely represented a microbial dysbiosis induced by a pathogen.

It is oftentimes difficult to establish or define what constitutes a ‘dysbiosis’ in a microbiome association study (Brüssow, 2020); however, the collection of samples prior to, during, and after challenge by a known pathogen does allow us to - at a minimum - define individual study participant ‘eubiotic’ states as those observed prior to challenge (pre-challenge), and the induction of a dysbiotic state through the successful colonization of *C. jejuni* and development of campylobacteriosis during the inpatient period, which aligns with the definition of dysbiosis proposed by Levy et al. (2017). Upon *C. jejuni* challenge, compositional differences between treatment groups emerged. A significantly different microbial community composition was observed between study participants with campylobacteriosis who received rifaximin *versus* placebo (**Figure 3B**). However, these differences were small (*i.e.*, an apparent low effect, p = 0.001, R^2^ = 0.042) and should not be over-interpreted. Greater differences were observed in microbial diversity (*i.e.*, alpha diversity), with rifaximin recipients who developed campylobacteriosis having significantly lower Shannon diversity than placebo recipients with campylobacteriosis during the inpatient period (*i.e.*, after *C. jejuni* challenge) (**Figure 4**). Reduced alpha diversity has been previously observed following rifaximin treatment in common variable immunodeficiency (CVID) patients (Jørgensen et al., 2019). While a significant decline in alpha diversity was identified during the inpatient period, there exists good evidence that rifaximin is well-tolerated over long-term use for patients with irritable bowel syndrome, and this change may not be clinically relevant (Mullen et al., 2014). Still, in our study, microbial taxa were lost or decreased in relative abundance within the rifaximin-treated study participants. Of the taxa identified that were significantly differentially abundant between treatment groups during the inpatient period, only the *Bacteroides* were in significantly greater relative abundance within the rifaximin-treated group (**Figures 2** and **5**); all other identified differential taxa were low in relative abundance (**Figure 5**), perhaps not influencing the clinical outcomes. Because differences were assessed as relative abundance, it is most likely that the loss of numerous, low abundance taxa resulted in the observed relative increase in the abundance of *Bacteroides.* Also of note, study participants who previously participated in enteric CHIM trials had significantly lower alpha diversity upon *C. jejuni* challenge, despite having non-significant differences in Shannon diversity at baseline. After challenge, prior CHIM participants had significantly higher Shannon diversity, seemingly ‘rebounding’ to a greater degree than those study participants participating in their first CHIM. More research is needed to determine whether this is an artifact of the study design or a true signal of increased resiliency in host microbiomes after repeated enteric pathogen exposure. Study participants who previously enrolled in a CHIM had no obvious differences in clinical outcomes during the trial (Rimmer et al., 2018), and if associated with prior CHIM participation, the inciting event (challenge or antibiotic treatment) could not be differentiated here. Future work including both retrospective meta-analyses of the available literature as well as incorporating the microbiome-based techniques presented here into future cohort-based studies are needed to fully integrate the host innate immune response, the microbial community response to both challenge and antibiotic treatment, and the effect of multiple CHIM trials over an extended duration.

We examined whether gut communities returned to their pre-challenge states or if an observable dysbiosis persisted. While the observation period was short, study participants largely did not return to their pre-challenge states, as indicated by significant differences in community assemblage and lower diversity in all study participant samples. Beta diversity was significantly different during the post-discharge period relative to the pre-challenge samples (adonis p = 0.002, R^2^ = 0.040), indicating a minor but statistically significant shift in study participant microbiomes, in which numerous genera were significantly differentially abundant (**Figure 5**). The genus *Bacteroides* significantly increased in relative abundance in post-discharge samples, driven mostly by the removal of numerous low-abundance taxa (as evidenced by the lower alpha diversity observed post-discharge, **Figure 4**) as well as a loss of the abundant genus *Faecalibacterium* (**Figure 2**). This depletion is almost certainly due to the dual antibiotic treatment with azithromycin and ciprofloxacin received by all study participants. This is supported by the fact that most of the observed taxa that declined in abundance during this period, including *Faecalibacterium* and Ruminicoccaceae, are known to be susceptible to ciprofloxacin (Stewardson et al., 2015). Whether or not this post-CHIM microbiome persists cannot be ascertained from this dataset. Previous work suggests that this new *Bacteroides*-rich, lower diversity microbiome will persist within study participants; however, significant dietary shifts can allow for the observed microbiota to return to their pre-challenge state (David et al., 2014). Increased duration of follow-up sampling, as well as either a study in which only ciprofloxacin and azithromycin are administered without challenge by an enteric pathogen (*e.g.* study participants recruited while receiving these antibiotics for treatment outside of enteric pathogens), could assist in answering this question.

There were numerous individual ASVs/taxa that were significantly differentially abundant when observing a shift from the pre-challenge ‘eubiotic’ state to the inpatient ‘dysbiotic’ state that may contribute to an increased risk for campylobacteriosis. *C. jejuni* was significantly increased in relative abundance in inpatient samples (as expected, as no study participant had been previously exposed to the pathogen), but several other microorganisms were also significantly differentially abundant, including unclassified members of the Ruminococcaceae (UCG-004 and Ruminoclostridium 5) and Lachnospiraceae (UCG-004, UCG-010, and *Lachnoclostridium*). Unclassified lineages of the Ruminococcaceae have been previously identified in uncontrolled travelers’ diarrhea studies in military populations (Stamps et al., 2020; Walters et al., 2020), but additional experimentation is needed to discern their metabolic capability and if they could be classified as ‘pathobionts’, or if their identification is merely coincidental (Chow and Mazmanian, 2010; Levy et al., 2017). Three taxa, *Butyricicoccus, Parasutterella*, and *Agathobacter*, were significantly more abundant in pre-challenge samples for study participants who recrudesced asymptomatically. Both *Parasutterella* and *Agathobacter* have previously been associated with bile acid cycling, and *Agathobacter* in the production of butyrate, which is generally associated with positive gut function (Feng et al., 2019; Ju et al., 2019); and so, their increased abundance in recrudescent study participants is surprising. *Campylobacter* spp. have previously been detected in the gallbladders of nonhuman species (Ertaş et al., 2003) and in rare cases associated with cholecystitis (inflammation of the gallbladder) within humans (Dakdouki et al., 2003). The association with the gallbladder and bile acid cycling should be explored further to understand if any association exists between *Parasutterella, Campylobacter*, and recrudescence. *Butyricicoccus* was identified as having a protective effect in broiler chickens exposed to *Campylobacter*, and so its identification as a potential risk factor in recurrent campylobacteriosis in humans warrants further study to identify how, or if, this microorganism may contribute to recurrent disease (Eeckhaut et al., 2016).

Alongside understanding how the human gut changes during infection, identification of specific microbial taxa that reduce campylobacteriosis risk is of keen interest. When comparing pre-challenge samples of study participants who did or did not develop campylobacteriosis, we were able to identify five genera that were differentially abundant (**Supplemental Figure S6B**). Two genera were Ruminiclostridium group species, previously reported to be associated with healthy control study participants in an association study of diabetes mellitus (Kuang et al., 2017). Another, Defluviitaleaceae UCG-011, was previously associated with increased relative abundance and *Campylobacter* infection in mice fed a zinc-deficient diet; though this increase was only noted post-infection, not before (Giallourou et al., 2018). Lastly, one of the most common gut commensal genera, *Bacteroides*, was also found to be significantly elevated in study participants with campylobacteriosis. As a secondary confirmatory test, a random forests model also identified *Bacteroides* ASVs as top predictors of whether a sample originated from study participants with or without campylobacteriosis. A recent study noted that the normally commensal *Bacteroides vulgatus* can co-metabolize sugars with *C. jejuni*, increasing the likelihood of successful colonization and infection (Garber et al., 2020); nonetheless, the described sugar, L-fucose, cannot be metabolized by *C. jejuni* CG8421 because this strain is missing the Cj0480-Cj0489c locus involved in fucose transport and metabolism, and so the exact link between *C. jejuni* and *Bacteroides* species requires additional study. One *Bacteroides* ASV was identified, ASV1, which was only sparsely present within study participants who developed campylobacteriosis, but present in all four study participants without disease (**Supplemental Table S2**). A potential explanation is that strain-level variation in the *Bacteroides* may confer differences in substrate utilization and sharing that could confer resistance to enteric infection, a possibility that requires additional experimentation. Taking the differential abundance data and observations in increased alpha diversity in study participants who did not develop campylobacteriosis, it appears that an increased host gut diversity and composition may be protective against campylobacteriosis. Conversely, a significantly decreased alpha diversity and a general lack of the identified marker taxa may increase the risk for enteric illness following pathogen exposure.

The *C. jejuni* CHIM revealed potential correlations between certain study participants seemingly protected from campylobacteriosis and the composition of their gut microbiome. Our work offers tantalizing clues to the potential for a protective microbiome against enteric infection. Future work using humanized animal models may advance the establishment of a causative link for potentially ‘protective’ microbial communities within the gut. Enteric diseases continue to be a leading cause of morbidity and mortality globally, particularly in LMICs (Murray et al., 2012; Olson et al., 2019). It should be noted that our work is representative of adult individuals living in a high-income country and does not fully represent the risks of *C. jejuni* infection posed to children in LMICs. Additionally, the identification and elucidation of a ‘host factor profile’, with the microbiome as only one of the many factors, should greatly expand our ability to understand not only the microbial but also the host contribution to enteric pathogen resistance or susceptibility. Specific factors could include demographic variables that may influence food choices or availability, body mass index, pre-challenge immune measures such as IgG, strain-specific IFN-gamma, fecal inflammatory biomarkers, and pathogen-specific fecal IgA. Once identified, each of these could be fed into a multi-omic analysis to provide a more holistic picture and begin to move beyond simple correlations. Our work provides an initial look into gut microbiome shifts observed during a controlled enteric infection and following antibiotic treatment; ongoing and future studies stand ready to potentially relieve humanity of one of its longest-standing diseases, diarrhea.

## Regulatory Approval

The clinical trial from which data and samples were obtained was registered with ClinicalTrials.gov on 29 October 2014 (NCT02280044), and was reviewed and approved by the Naval Medical Research Center and Western Institutional Review Boards in compliance with all applicable local, federal, and Department of Defense regulations governing the protection of human subjects. The protocol under which this work was performed (IDCRP-079) was reviewed and approved by the Institutional Review Board at the Infectious Disease Clinical Research Program (IDCRP).

The contents of this publication are the sole responsibility of the author(s) and do not necessarily reflect the views, opinions, or policies of the Uniformed Services University of the Health Sciences (USUHS), The Henry M. Jackson Foundation for the Advancement of Military Medicine, Inc. (HJF), the Department of Defense (DoD), the Departments of the Army, Navy, or Air Force, the UK Ministry of Defence, Naval Medical Research Center (NMRC), Air Force Research Laboratory (AFRL), or US Government. Mention of trade names, commercial products, or organizations does not imply endorsement by the US Government.

Authors are military service members or federal/contracted employees of the US Government. This work was prepared as part of official duties. Title 17 USC § 105 provides that `Copyright protection under this title is not available for any work of the US Government.’ Title 17 USC § 105 defines a US Government work as work prepared by a military service member or employee of the US Government as part of that person’s official duties.

## Data Availability Statement

The datasets presented in this study can be found in online repositories. The names of the repository/repositories and accession number(s) can be found below: https://www.ncbi.nlm.nih.gov/bioproject/PRJNA727025 Accession numbers: SRR14412871 to SRR14413158.

## Author Contributions

BS processed experimental data and wrote the manuscript. JK participated in stool collection, aliquoting, archiving, DNA extraction, and processing for sequencing. SI provided the clinical data for analysis, assisted with ethical review processes, and reviewed and edited the manuscript. MS critically reviewed and edited the manuscript. CH participated in the clinical trial design and execution. KT participated in the clinical trial conduct, and reviewed and edited the manuscript. DS and DT participated in the clinical trial design and execution, and reviewed and edited the manuscript. AM and RL were responsible for the custody, receipt, and distribution of stool specimens from the clinical study, and reviewed and edited the manuscript. JR established the protocol to ensure sample collection, oversaw data and sample collection, and reviewed and edited the manuscript. CP conceived of and designed the clinical trial, developed the sample collection plans, provided the clinical data for analysis, assisted with the scientific and ethical review processes, and reviewed and edited the manuscript. MG critically reviewed and edited the manuscript. FP designed and supervised the stool collection plan, participated in stool collection, aliquoting, and archiving, selected samples for sequencing, established the collaboration for data analysis, and reviewed and edited the manuscript. All authors contributed to the article and approved the submitted version.

## Funding

This work (IDCRP-079) was conducted by the Infectious Disease Clinical Research Program (IDCRP), a Department of Defense (DoD) program executed by the Uniformed Services University of the Health Sciences (USUHS), through a cooperative agreement with The Henry M. Jackson Foundation for the Advancement of Military Medicine, Inc. (HJF), a memorandum of agreement between NMRC and IDCRP (NMR-9791/PMB.15.232), and between NMRC and the 711^th^ Human Performance Wing/Airman Systems Directorate (NMR-10423). Funding for the 16S rRNA sequencing was performed under Navy Work Unit 6000.RAD1.DA3.A0308. This project has been funded in whole, or in part, with federal funds from the Military Infectious Diseases Research Program (MIDRP).

## Conflict of Interest

BS is an employee of UES, Inc. JK, SI, AM, and RL are (or were at the time of the study) employees of the Henry M. Jackson Foundation for the Advancement of Military Medicine.

The remaining authors declare that the research was conducted in the absence of any commercial or financial relationships that could be construed as a potential conflict of interest.

## Publisher’s Note

All claims expressed in this article are solely those of the authors and do not necessarily represent those of their affiliated organizations, or those of the publisher, the editors and the reviewers. Any product that may be evaluated in this article, or claim that may be made by its manufacturer, is not guaranteed or endorsed by the publisher.

## Supplementary Material

Supplementary Table 1 Metadata file used to generate plots and analysis within DADA2 and Phyloseq. Study participant data are taken from our previous study (Rimmer et al., 2018). This data file includes study participant metadata such as age, sex, treatment group, symptoms, as well as a column to define if study participants were previously enrolled in other CHIMs.

Supplementary Table 2 Additional per-sample library read counts after QC and ASV generation and minimum, maximum, and mean library size **(A)**. Detailed ASV table including per-sample ASV abundances and taxonomy of each ASV **(B)**.

Supplementary Table 3 Table of the top 50 ASVs sorted by a decrease in mean accuracy of the generated random forest model. ASV taxonomy is given alongside mean Gini decrease.

Supplementary Figure 1 Rarefaction curves for all samples, grouped by study participant. The number of sequence reads is along each plot x-axis, and the number of observed ASVs is shown on the y-axis.

Supplementary Figure 2 Principal component analysis of all samples from this study (triangles) compared to those taken from the Broad Institute-OpenBiome Microbiome Library (circles) (Poyet et al., 2019). Samples from this study are shown grouped by study period (e.g., pre-challenge, inpatient, post-antibitoic administration, and post-discharge).

Supplementary Figure 3 Heatmap showing samples grouped by study period (e.g., pre-challenge, inpatient, post-antibitoic administration, and post-discharge), with the most abundant ASVs shown. Gray squares indicate an ASV was not detected.

Supplementary Figure 4 Extended heatmap showing all samples, with the 50 most abundant ASVs shown. Samples are grouped by study period (e.g., pre-challenge, inpatient, post-antibitoic administration, and post-discharge).

Supplementary Figure 5 Principal coordinate analysis (PCoA) of samples from the pre-challenge (blue) to post-discharge period (red). PCoA was generated from a weighted UniFrac distance matrix.

Supplementary Figure 6 Additional differential abundance analyses including comparisons between treatment groups during the pre-challenge period **(A)**, between study participant samples during the pre-challenge period from study participants who did and did not develop campylobacteriosis **(B)**, and finally samples taken after antibiotic administration during the inpatient period in study participants who did and did not develop campylobacteriosis **(C)**.
