# Supplementary figures and images for: Exploring Changes in the Host Gut Microbiota During a Controlled Human Infection Model for Campylobacter jejuni
Source: Front Cell Infect Microbiol. 2021 Aug 31;11:702047. doi: 10.3389/fcimb.2021.702047 (PMC8439579; doi:10.3389/fcimb.2021.702047)

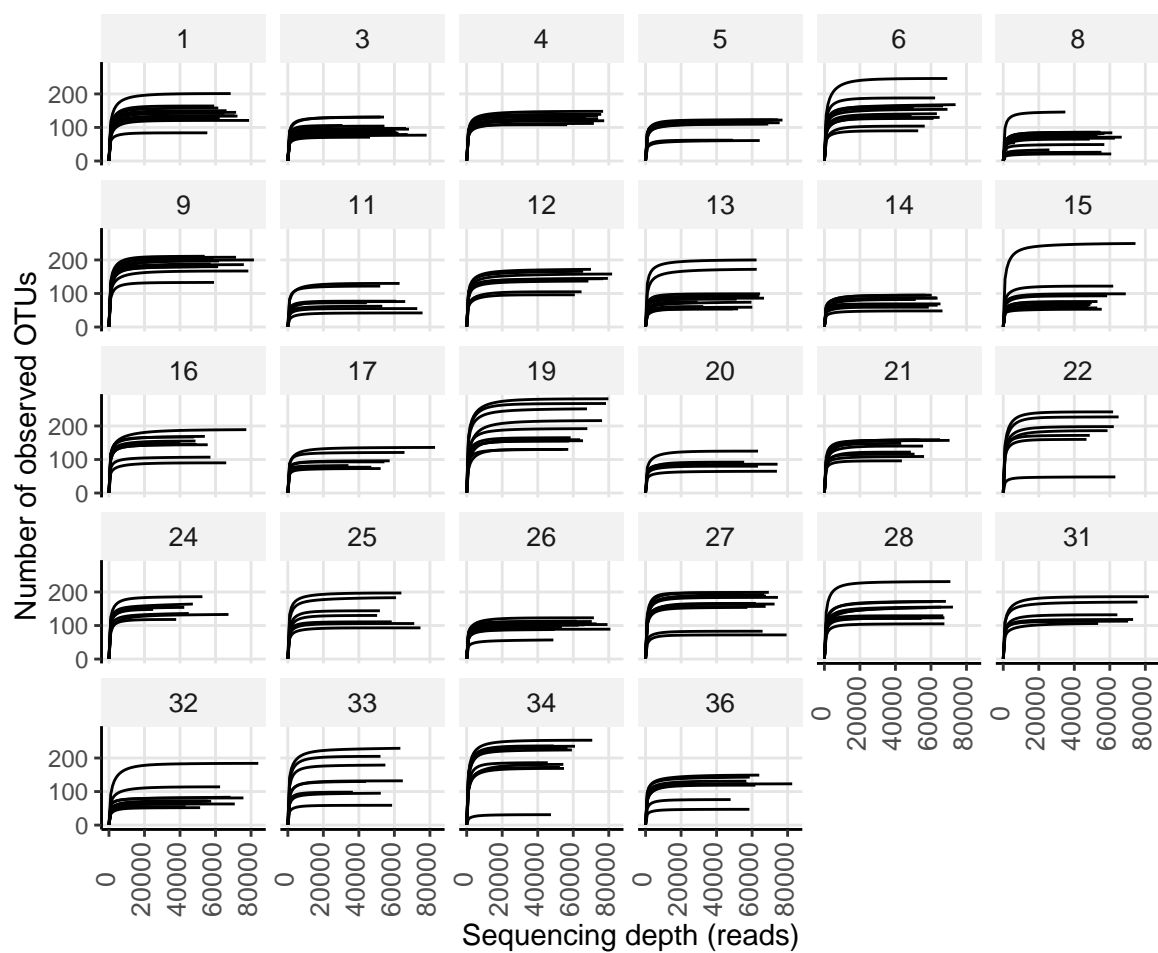

Supplement: Supplementary Figure 1 — Rarefaction curves for all samples, grouped by study participant. The number of sequence reads is along each plot x-axis, and the number of observed ASVs is shown on the y-axis. [file DataSheet_1.pdf]

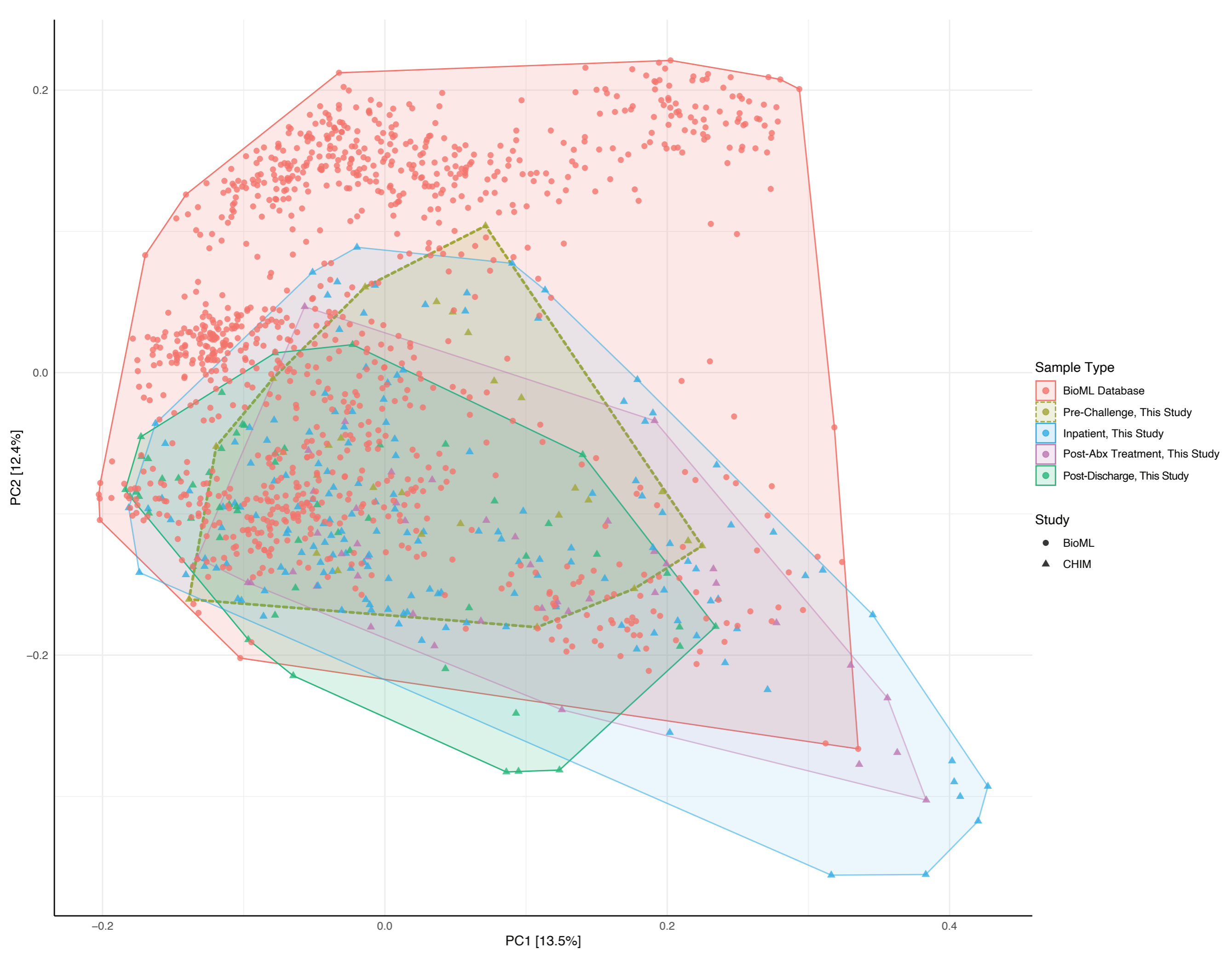

Supplement: Supplementary Figure 2 — Principal component analysis of all samples from this study (triangles) compared to those taken from the Broad Institute-OpenBiome Microbiome Library (circles) (Poyet et al., 2019). Samples from this study are shown grouped by study period (e.g., pre-challenge, inpatient, post-antibitoic administration, and post-discharge). [file DataSheet_2.pdf]

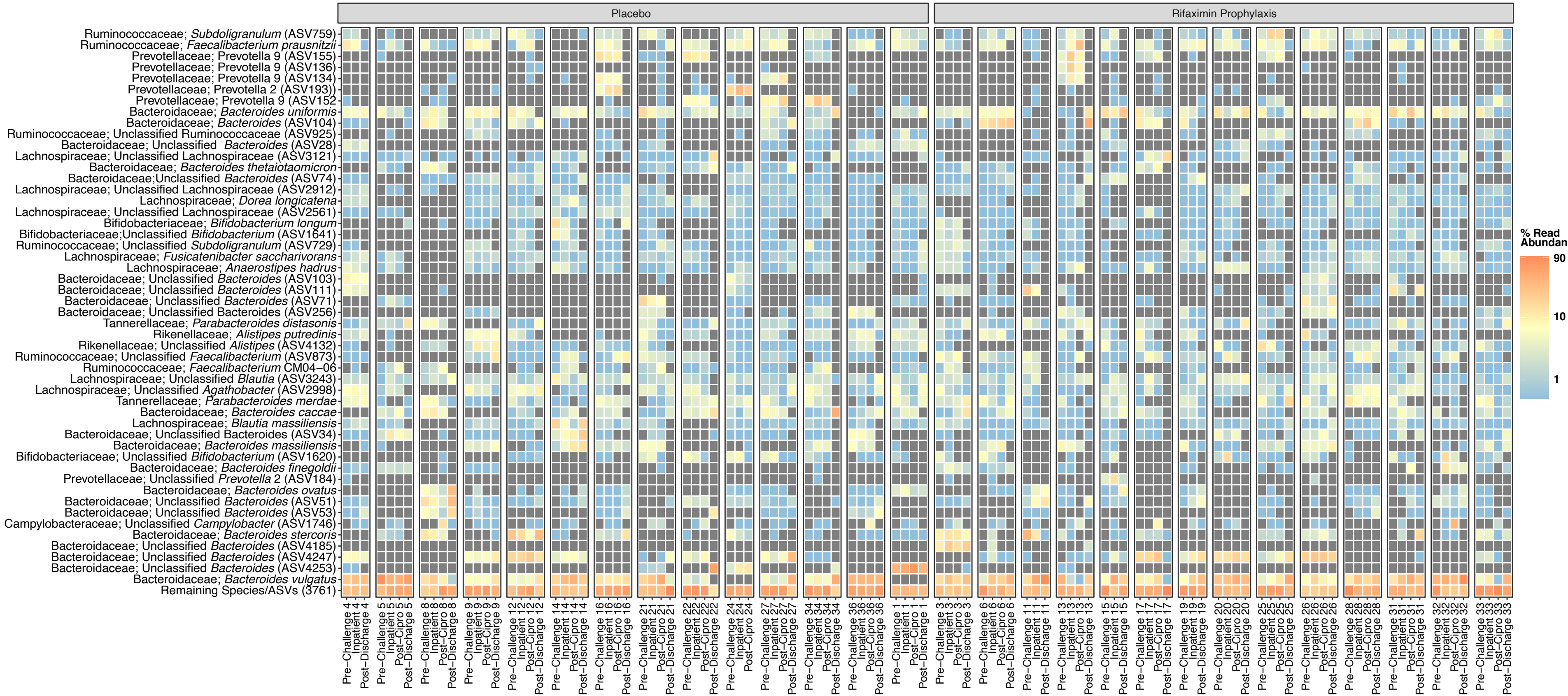

Supplement: Supplementary Figure 3 — Heatmap showing samples grouped by study period (e.g., pre-challenge, inpatient, post-antibitoic administration, and post-discharge), with the most abundant ASVs shown. Gray squares indicate an ASV was not detected. [file DataSheet_3.pdf]

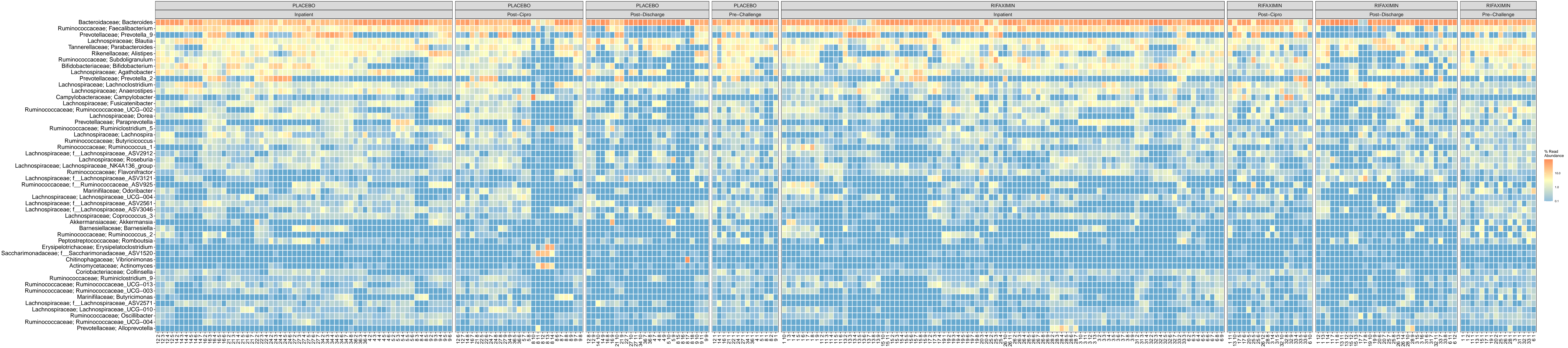

Supplement: Supplementary Figure 4 — Extended heatmap showing all samples, with the 50 most abundant ASVs shown. Samples are grouped by study period (e.g., pre-challenge, inpatient, post-antibitoic administration, and post-discharge). [file DataSheet_4.pdf]

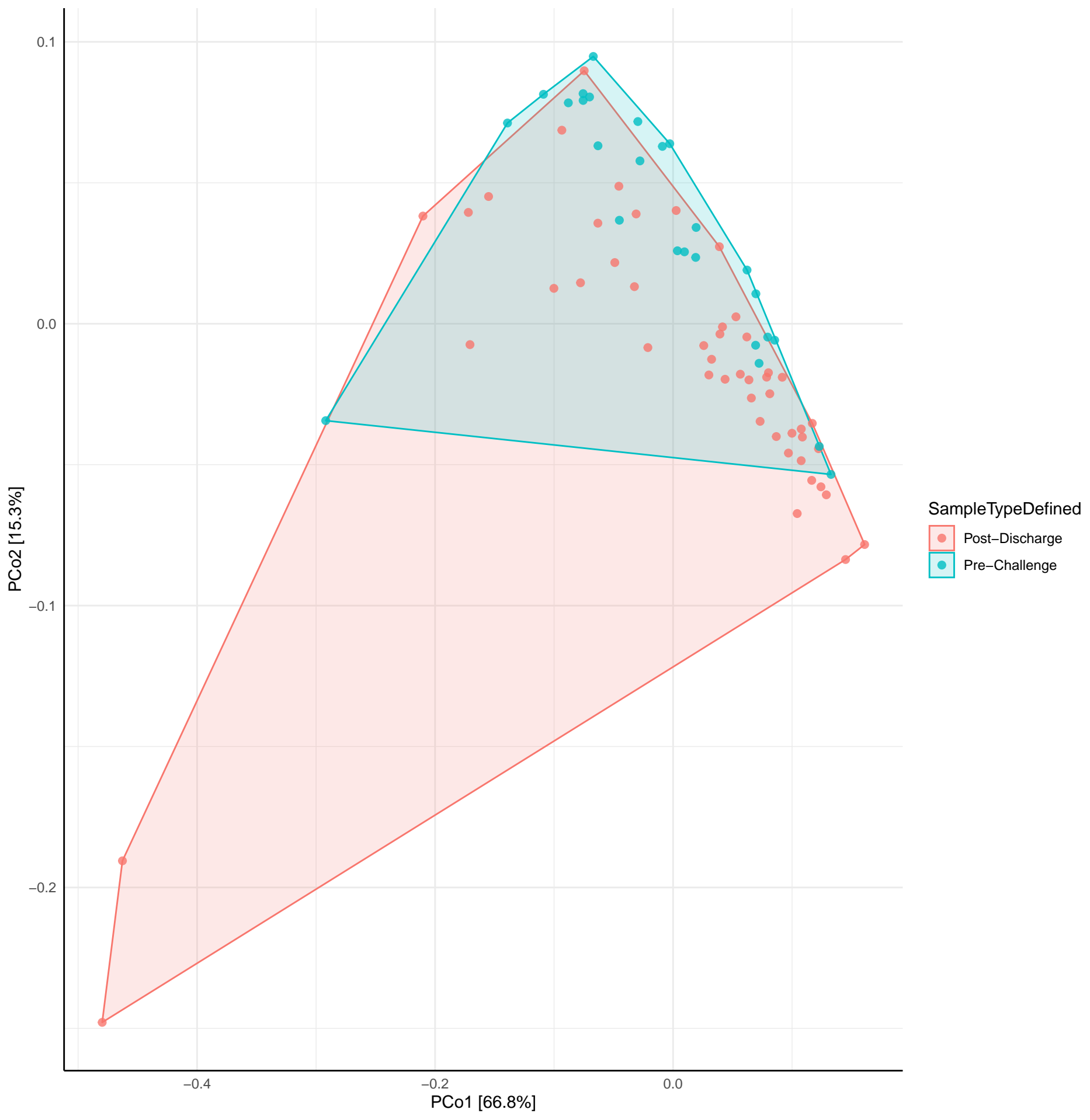

Supplement: Supplementary Figure 5 — Principal coordinate analysis (PCoA) of samples from the pre-challenge (blue) to post-discharge period (red). PCoA was generated from a weighted UniFrac distance matrix. [file DataSheet_5.pdf]

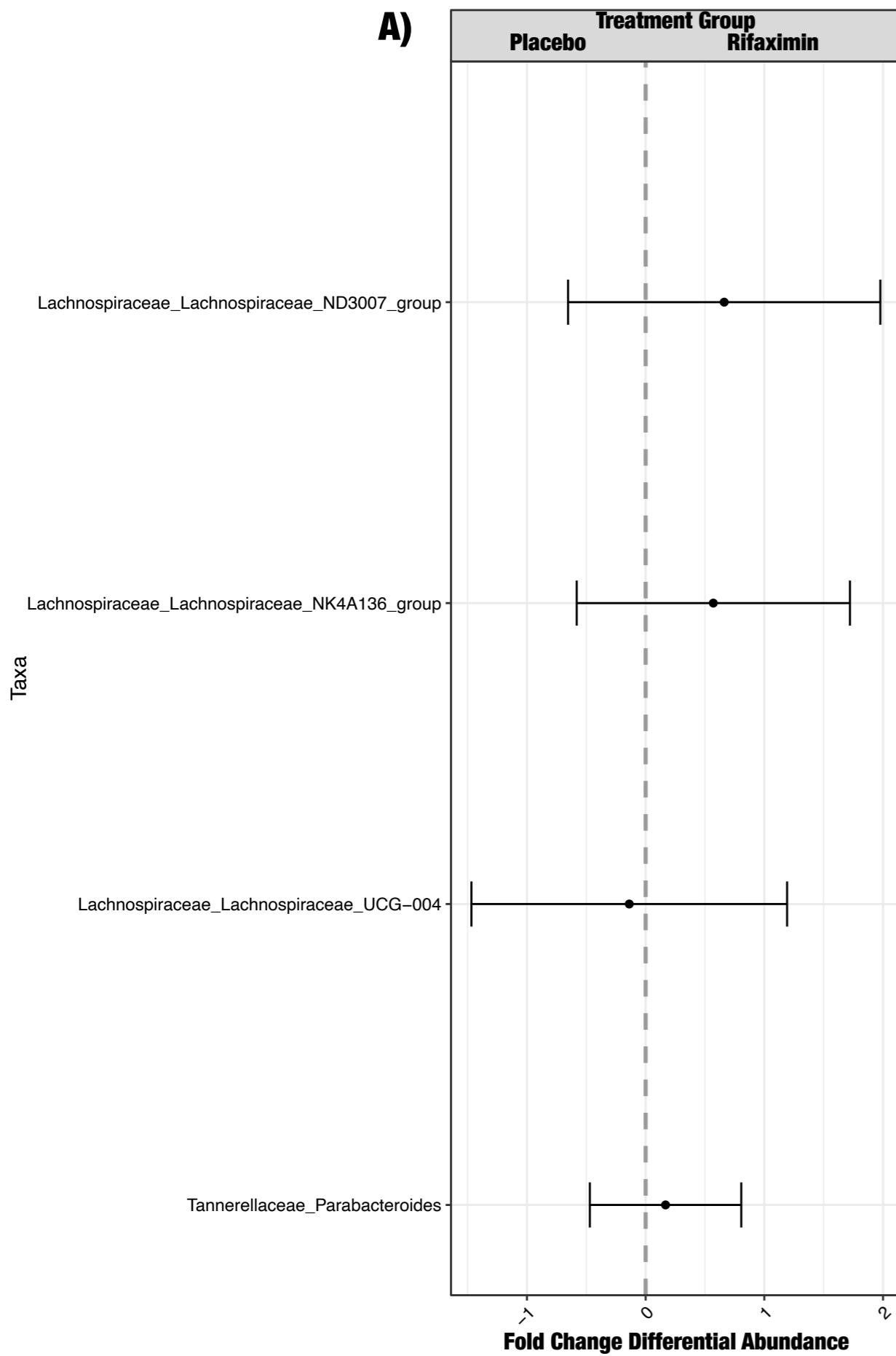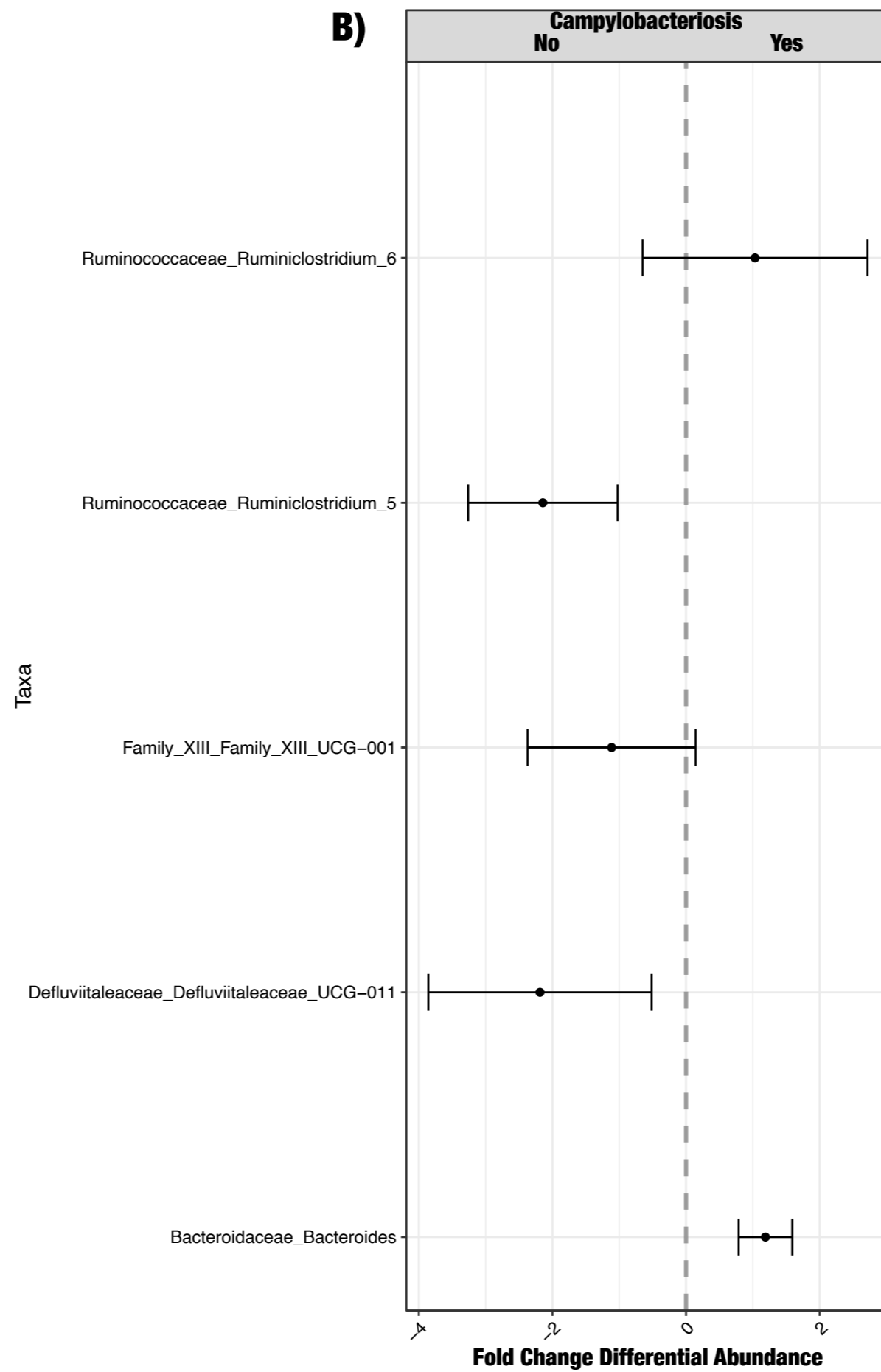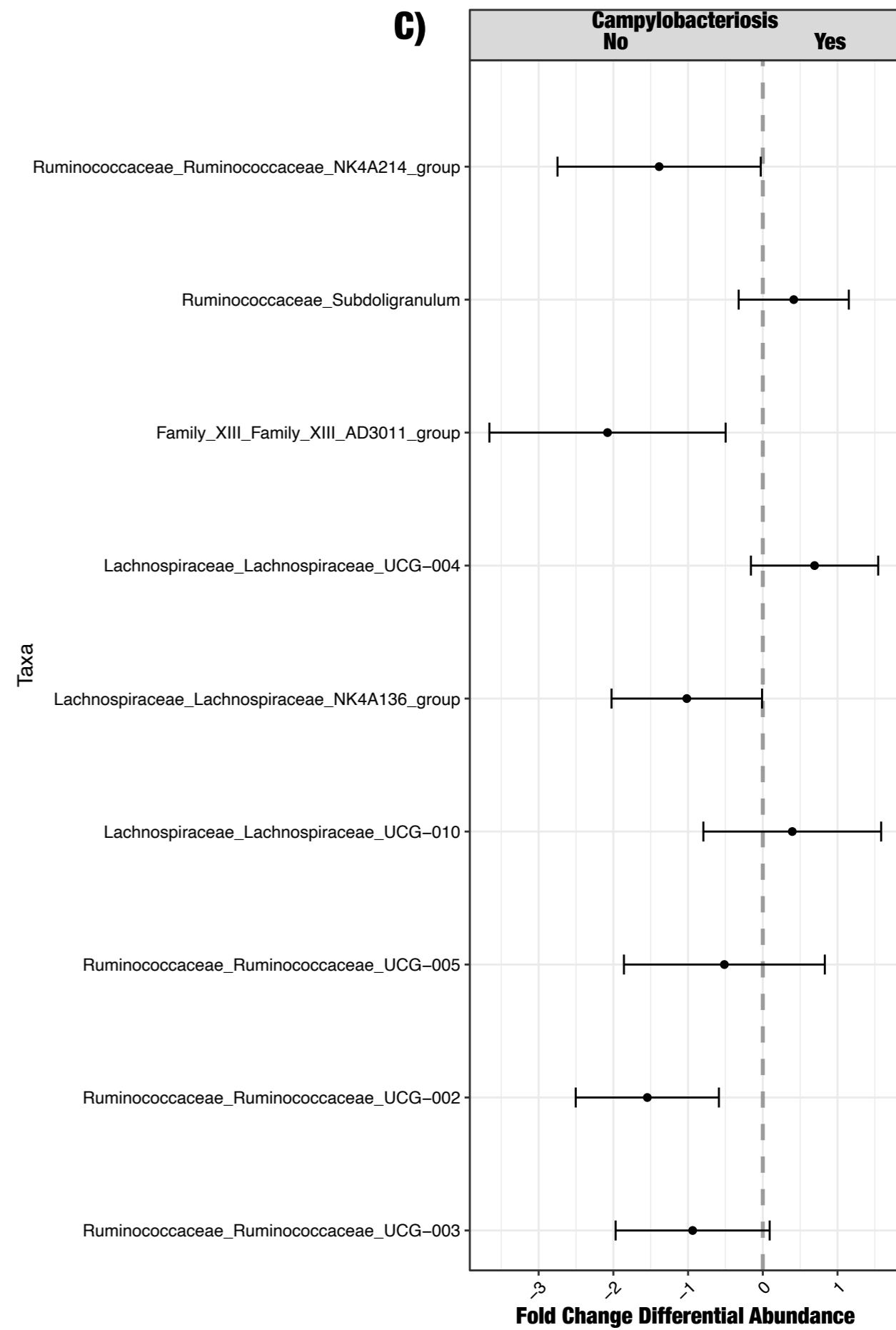

Supplement: Supplementary Figure 6 — Additional differential abundance analyses including comparisons between treatment groups during the pre-challenge period (A), between study participant samples during the pre-challenge period from study participants who did and did not develop campylobacteriosis (B), and finally samples taken after antibiotic administration during the inpatient period in study participants who did and did not develop campylobacteriosis (C). [file DataSheet_6.pdf]
